# Discrepancies of video head impulse test results in patients with idiopathic sudden sensorineural hearing loss with vertigo and vestibular neuritis

**DOI:** 10.3389/fnins.2023.1102512

**Published:** 2023-04-17

**Authors:** Yingzhao Liu, Yangming Leng, Renhong Zhou, Jingjing Liu, Hongchang Wang, Kaijun Xia, Bo Liu, Hongjun Xiao

**Affiliations:** Department of Otorhinolaryngology-Head and Neck Surgery, Union Hospital, Tongji Medical College, Huazhong University of Science and Technology, Wuhan, China

**Keywords:** vestibular neuritis, idiopathic sudden sensorineural hearing loss with vertigo, vestibulo-ocular reflex, video head impulse test, saccade, gain

## Abstract

**Objective:**

Sudden sensorineural hearing loss with vertigo (SHLV) and vestibular neuritis (VN) remain frequent causes of acute vestibular syndrome (AVS). The aim of study was to compare the results of video head impulse test (vHIT) in patients with SHLV and VN. The characteristics of high-frequency vestibule-ocular reflex (VOR) and the differences of the pathophysiological mechanisms underlying these two AVS were explored.

**Methods:**

Fifty-seven SHLV patients and 31 VN patients were enrolled. vHIT was conducted at the initial presentation. The VOR gains and occurrence of corrective saccades (CSs) of anterior, horizontal, and posterior semicircular canals (SCCs) in two groups were analyzed. Pathological vHIT results refer to impaired VOR gains and presence of CSs.

**Results:**

In SHLV group, pathological vHIT results was most prevalent in the posterior SCC on the affected side (30/57, 52.63%), followed by horizontal (12/57, 21.05%) and anterior SCC (3/57, 5.26%). In VN group, pathological vHIT preferentially affected horizontal SCC (24/31, 77.42%), followed by anterior (10/31, 32.26%) and posterior SCC (9/31, 29.03%) on the affected side. As for anterior and horizontal SCC on the affected side, the incidences of pathological vHIT results in VN group were significantly higher than those in SHLV group (*β* = 2.905, *p* < 0.01; *β* = 2.183, *p* < 0.001). There were no significant differences in the incidence of pathological vHIT result in posterior SCC between two groups.

**Conclusion:**

Comparison of vHIT results in patients with SHLV and VN revealed discrepancies in the pattern of SCCs impairments, which may be explained by different pathophysiological mechanisms underlying these two vestibular disorders presenting as AVS.

## 1. Introduction

Acute vestibular syndrome (AVS) refers to a series of acute disorders with vertigo or dizziness as the main symptom, which may be accompanied by clinical manifestations such as nausea, vomiting, unsteadiness or nystagmus (Tarnutzer et al., 2011). Common AVS include vestibular neuritis (VN), vertigo caused by central cerebrovascular injury, etc. (Tarnutzer et al., 2011). Idiopathic sudden sensorineural hearing loss (ISSNHL) is defined as sudden hearing loss more than 30 dB in three consecutive frequencies, occurring within 72 h with no identifiable cause despite extensive surveys (Stachler et al., 2012). Among them, the acute vertigo attack may accompany hearing loss concurrently or occur successively in more than 30% of patients, which is also known as sudden sensorineural hearing loss with vertigo (SHLV) (Yu and Li, 2018). The etiologies of ISSNHL or SHLV remains unclear. Yet hypotheses include vascular disturbance, viral inflammation, autoimmune response, neoplasms, trauma, ototoxicity, developmental anomalies, metabolic factors, and psychogenic disorders (Koc and Sanisoglu, 2003). VN is an acute vestibular disorder characterized by vertigo, nausea and vomiting, without cochlear symptoms such as hearing loss or tinnitus (Jeong et al., 2013). The etiology of VN is generally believed to be vestibular nerve dysfunction caused by viral infection. Both SHLV and VN can manifest as AVS, whereas the results of vestibular tests differed between them (Jung et al., 2012). Jung et al. (2012) showed that there were no differences in the duration of spontaneous nystagmus (SN), duration of vertigo and the canal paresis (CP) value in caloric test between patients with SHLV and VN, but the abnormal rate of cervical vestibular evoked myogenic potentials (cVEMP) in VN patients was considerably lower than those in SHLV patients.

Video head impulse test (vHIT) is a newly developed instrumental vestibular test, which evaluates the angular vestibulo-ocular reflex (VOR) function of individual semicircular canals (SCCs) at high frequency. Normally, when head rotates rapidly, eyes will rotate in the opposite direction at same velocity through adjustment of VOR system to maintain gaze stabilization. The VOR gain value calculated by the ratio of eye movement to head movement was close to 1.0 in healthy subjects. In patients with peripheral vestibular hypofunction, VOR function is damaged with gain reduction and presence of corrective saccades (CSs), which may occur during (covert) or after the head impulse (overt). To the best of our knowledge, until now, few studies have compared the differences of vHIT findings in patients with SHLV and VN. In one retrospective chart review, Yao et al. demonstrated that the gains of anterior and horizontal SCC in VN patients were significantly lower than those in SHLV subjects (Yao et al., 2018). To date, no study explored the differences of vHIT findings between the SHLV and VN in terms of gain and CSs simultaneously.

In this retrospective study, we compared the value of VOR gain and occurrence of CSs between patients with SHLV and VN. We aimed to investigate the lesion pattern of angular VOR in each SCC in these two vestibular disorders presenting as AVS, and to further explore the underlying pathophysiological mechanism.

## 2. Materials and methods

### 2.1. Study population

A single-center retrospective chart review of patients suffering from acute onset of severe vertigo between October 2017 and August 2021 at the Department of Otolaryngology-Head and Neck Surgery, Union Hospital, Tongji Medical College, Huazhong University of Science and Technology, Wuhan, China was conducted.

Fifty-seven patients diagnosed as unilateral SHLV and 31 patients diagnosed as unilateral VN were included. The inclusion criteria were: (1) Patients with SHLV presented with acute onset of sensorineural hearing loss in one ear at three consecutive frequencies more than 30 dB HL within 72 h without an identifiable cause (unilateral ISSNHL), as well as acute vertigo attack within 24 h of the onset of hearing loss. The diagnosis of ISSNHL is established according to the clinical practice guideline of sudden hearing loss proposed by American Academy of Otolaryngology-Head and Neck Surgery (AAO-HNS) in 2012 (Stachler et al., 2012). (2) The patients with VN had sudden attack of rotatory vertigo without auditory symptoms. Peripheral vestibular impairment was confirmed by pathological caloric test and/or vHIT results. (3) Audiometry, vHIT, and caloric test were performed at the time of initial assessment.

Exclusion criteria were: (1) additional concurrent vestibular disorders (Ménière’s disease, Ramsay Hunt’s syndrome, vestibular migraine, benign paroxysmal positional vertigo, etc); (2) recurrent acute low-tone sensorineural hearing loss; (3) middle ear infections (otitis media, mastoiditis, etc); (4) middle or inner ear anomaly; (5) having received previous ear surgery or intratympanic injection; (6) retro-cochlear lesions; (7) disorders of central nervous system (cerebellar infarction, multiple sclerosis, etc).

This study was conducted in accordance with the tenets of the Declaration of Helsinki. The study was approved by the Ethical Committee of Union Hospital, Tongji Medical College, Huazhong University of Science and Technology, Wuhan, China.

### 2.2. Examination procedure

For all patients, medical history was collected in detail, and pure tone audiometry, caloric test and vHIT were performed. Routine imaging evaluation include non-contrast magnetic resonance imaging (MRI) of the inner ear, mainly to rule out retro-cochlear pathology. In case of suspicious lesions, contrast-enhanced MRI was warranted.

#### 2.2.1. Caloric test

Bithermal caloric response was measured using infrared videonystagmography (Visual Eyes VNG, Micromedical Technologies, Chatham, IL, United States). Patients lay supine with their head and upper trunk elevated at 30°. Each ear was inflated alternately with a constant flow of air, with the temperature for warm or cool stimulation set at 50°C and 24°C, respectively. The duration of each caloric stimulation lasted 60 s. Upon each inflation, the maximum slow phase velocity (SPV_max_) of caloric nystagmus was measured, and the CP was calculated by using the Jongkees’ formula. In this study, interaural asymmetry of the caloric nystagmus ≥25% was considered significant, indicating an abnormal caloric response in the horizontal SCC. According to the published criteria, if the summated SPV_max_ of the nystagmus was less than 20°/s under 4 stimulation conditions, the caloric response is believed to indicate bilateral vestibular hypofunction. In this case, ice water irrigation (4°C, 1.0 ml) would be used to confirm the caloric unresponsiveness.

#### 2.2.2. vHIT

vHIT was conducted using an ICS Impulse system (GN Otometrics, Denmark) consistent with the manufacturer’s instructions by experienced technicians. Each patient wore a pair of lightweight, tightly-fitting goggles equipped with a small video oculography camera to record and analyze the eye movement. Patient was seated upright facing the wall 1.0 m away and was instructed to fixate a static target on the wall. A technician standing behind the patient manually delivered approximately 20 to 25 random, unpredictable and passive head impulses (amplitude: 5 ~ 15°, peak velocity: 150 ~ 250°/s, duration: 150–200 ms) in the plane of each SCC. Re-fixation CSs were categorized, against their appearance, as covert and overt. If the velocity of the CSs exceeded 50°/s, they were considered significant. In the present study, the cut-off values in VOR gain were 0.8 for the horizontal SCC and 0.7 for the vertical SCCs. A pathological vHIT result refers to the combination of decreased gain and the presence of re-fixation saccades.

### 2.3. Statistical analysis

Statistical analysis was performed using SPSS software (version R26.0.0.2). Continuous variables are presented as either mean ± standard deviation or median and quartiles. We used Cocnran’s *Q* test to analyze the incidence of pathological vHIT results of three SCCs on the affected side in two groups. Baseline data with normal distribution were compared using independent-sample t test between two groups. Nonnormally distributed data were compared using Mann–Whitney *U* test. Categorical variables were compared using Chi-square test. After comparison of baseline information, logistic regression analysis was performed to assess the impact of potential confounders. A significance level of *p <* 0.05 was used for the differences between the groups.

## 3. Results

### 3.1. Demographic characteristics in patients with SHLV and VN

The demographic characteristics of SHLV and VN patients were shown in Table 1. No differences in gender or affected side were found between two groups. The mean age and course duration in VN group were significantly higher than those in SHLV group. To make the results exact, we counted age and course duration as confounding factors and ruled out the influences in statistical analysis.

**Table 1:** Demographic and clinical characteristics of SHLV and VN patients.

		SHLV (*n* = 57)	VN (*n* = 31)	Test statistics	*p*-value
Gender (male/female)		24/33	19/12	*χ*^2^ = 2.958	0.085
Age (yr.)		40.84 ± 13.98	47.93 ± 13.26	*t* = −2.233	0.028
Course duration (days)		7 (4, 13.5)	20 (7, 40)	*U* = 1343.5	<0.001
Affected side (left/right)		31/26	13/18	*χ*^2^ = 1.245	0.265
SN (positive/negative)		27/30	26/5	*B* = −1.906	0.005
Caloric test	Abnormal rate	56/57 (98.25%)	30/31 (96.77%)	*B* = 1.807	0.128
	CP value (%)	43.29 ± 24.54	77.50 ± 23.71	*B* = 0.051	<0.001

SHLV, sudden sensorineural hearing loss with vertigo; VN, vestibular neuritis; SN, spontaneous nystagmus; CP, canal parasis.

### 3.2. vHIT results in patients with SHLV

**Figure 1:**
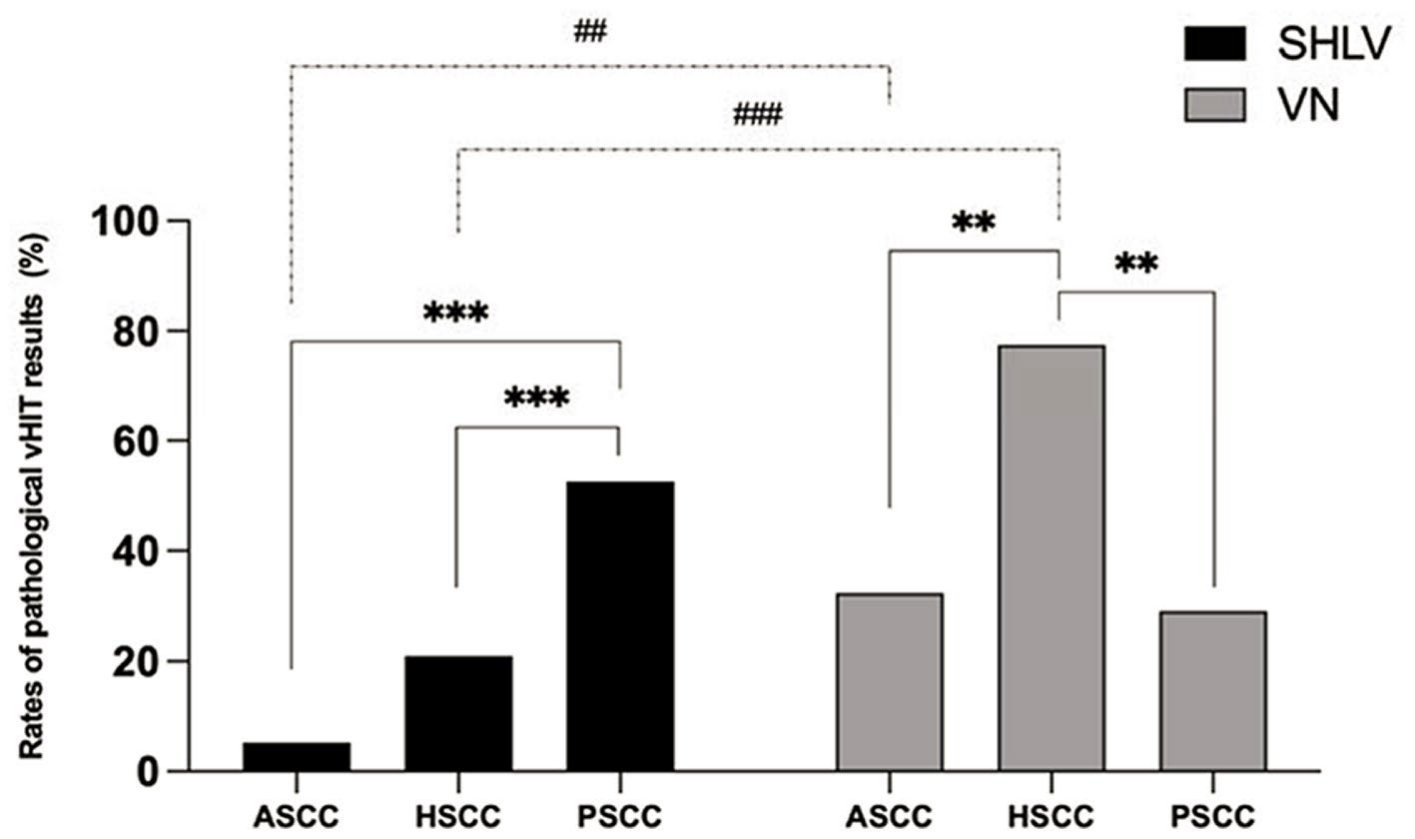
Comparison between the incidences of pathological video head impulse test (vHIT) results of anterior semicircular canal (ASCC), horizontal semicircular canal (HSCC) and posterior semicircular canal (PSCC) on the affected sides in sudden sensorineural hearing loss with vertigo (SHLV) patients and vestibular neuritis (VN) patients. **p < 0.01, ***p < 0.001, ^##^*p* < 0.01, ^###^*p* < 0.001. Dotted line: comparison between SHLV and VN group. Solid line: comparison between three semicircular canals (SCCs) within SHLV or VN group.

**Figure 2:**
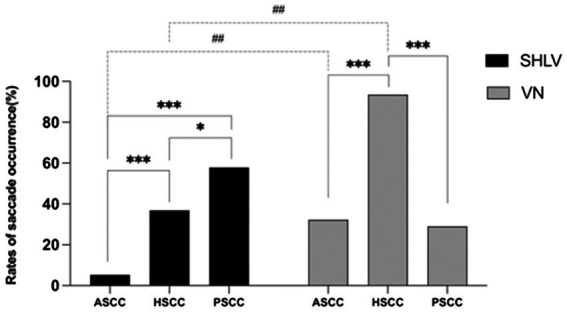
Comparison between the incidences of saccade occurrence in anterior semicircular canal (ASCC), horizontal semicircular canal (HSCC) and posterior semicircular canal (PSCC) on the affected sides in SHLV patients and VN patients. *p < 0.05, ***p < 0.001, ^##^*p* < 0.01. Dotted line: comparison between SHLV and VN group. Solid line: comparison between three semicircular canals within SHLV or VN group.

**Figure 3:**
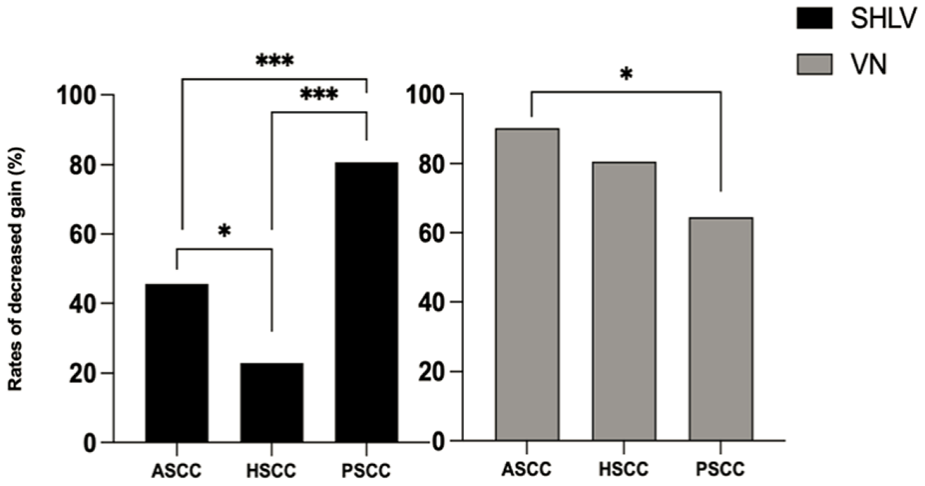
Comparison between the incidences of decreased VOR gain in anterior semicircular canal (ASCC), horizontal semicircular canal (HSCC) and posterior semicircular canal (PSCC) on the affected sides in SHLV patients and VN patients. *p < 0.05, ***p < 0.001.

As shown in Table 2, among SCCs on the affected side in SHLV group, pathological vHIT findings (Figure 1) and CSs (Figure 2) were most prevalent in posterior SCC (30/57, 52.63%; 33/57, 57.89%), followed by horizontal (12/57, 21.05%; 21/57, 36.84%) and anterior SCC (3/57, 5.26%; 3/57, 5.26%), and there were significant differences among three SCCs (*p*<0.05). Also, the incidence of decreased VOR gain was highest in posterior SCC (46/57, 80.7%), followed by anterior (26/57, 45.61%) and horizontal SCC (13/57, 22.81%), and the differences were significant when they compared with each other (*p* < 0.05; Figure 3). No SHLV patient showed pathological vHIT or CSs on the non-affected sides. All three SCCs on the affected sides had significantly lower VOR gain compared with non-affected sides (*p* < 0.05).

**Table 2:** Incidence of pathological vHIT results, frequency of CSs and VOR gain in three SCCs on the affected sides in patients with SHLV and VN.

	SHLV (*n* = 57)	VN (*n* = 31)	Test statistics (*β*)	*p*-value
Pathological vHIT results				
Anterior SCC	3/57 (5.26%)	10/31 (32.26%)	2.905	0.001
Horizontal SCC	12/57 (21.05%)	24/31 (77.42%)	2.183	<0.001
Posterior SCC	30/57 (52.63%)	9/31 (29.03%)	−0.714	0.209
Frequency of CSs				
Anterior SCC	3/57 (5.26%)	10/31 (32.26%)	2.905	0.001
Horizontal SCC	21/57 (36.84%)	29/31 (93.55%)	2.877	0.001
Posterior SCC	33/57 (57.89%)	9/31 (29.03%)	−0.969	0.089
VOR gain				
Anterior SCC	0.71 ± 0.19	0.46 ± 0.19	−6.088	<0.001
Horizontal SCC	0.93 ± 0.25	0.64 ± 0.20	−4.837	0.001
Posterior SCC	0.51 ± 0.20	0.61 ± 0.24	1.755	0.189

CSs, corrective saccades; vHIT, video head impulse test; VOR, vestibular ocular reflex; SCC, semicircular canal.

### 3.3. vHIT results in patients with VN

As shown in Table 2, among SCCs on the affected side in VN group, pathological vHIT results (Figure 1) and CSs (Figure 2) were most frequently observed in horizontal SCC (24/31, 77.42%; 29/31, 93.55%), followed by anterior (10/31, 32.26%; 10/31, 32.26%) and posterior SCC (9/31, 29.03%; 9/31, 29.03%), and there were significant differences between each two SCCs (*p*<0.01). The incidence of decreased VOR gain was highest in anterior SCC (28/31, 90.32%), followed by horizontal SCC (25/31, 80.65%) and posterior SCC (20/31, 64.52%), and significant difference was only observed between anterior and posterior SCC (*p* < 0.05. see in Figure 3). No pathological vHIT or CSs was detected in the non-affected ears in VN group. The VOR gain of the affected ears were significantly lower than that of the non-affected ears (*p* < 0.01).

### 3.4. Comparison of vHIT results on the affected side in patients with SHLV and VN

**Figure 4:**
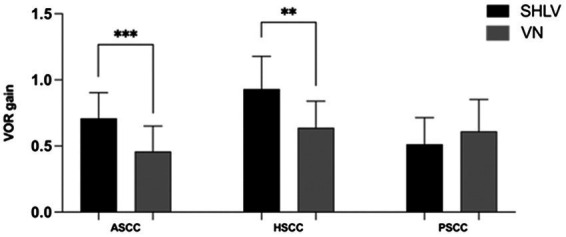
Comparison between the vHIT gain in anterior semicircular canal (ASCC), horizontal semicircular canal (HSCC) and posterior semicircular canal (PSCC) on the affected sides in SHLV patients and VN patients. **p < 0.01, ***p < 0.001.

Compared with SHLV patients, VN patients were more likely to have pathological vHIT results (Table 2; Figure 1), occurrences of CSs (Table 2, Figure 2), and lower VOR gains (Table 2; Figure 4) in anterior and horizontal SCCs. The incidence of pathological vHIT, frequency of CSs and VOR gain did not differ in posterior SCC between two groups (Table 2; Figures 1, 2, 4).

Moreover, as shown in Table 1, SN was more frequently found in VN patients (*β* = -1.906, *p* < 0.01. Table 1). Although the abnormal rates of caloric response did not differ between two groups (*β* = 1.807, *p* = 0.128), VN patients had significantly higher CP values (*β* = 0.051, *p* < 0.001). In this study, no patients showed bilateral vestibular hypofunction in caloric test.

## 4. Discussion

### 4.1. The lesion patterns of vHIT in SHLV patients

In this study, for the patients with SHLV, posterior SCC ipsilateral to hearing loss side was preferentially affected in terms of pathological vHIT results (52.63%), CSs occurrence (57.89%), and VOR gain reduction (80.7%). In a small cohort of patients with vertigo and sudden hearing loss (*n* = 27), Pogson et al. (2016) have demonstrated that 74% of patients had decreased VOR gain in posterior SCC. Meanwhile, only 41% of these patients exhibited decreased VOR gain in horizontal SCC and 30% in anterior SCC. Furthermore, Murofushi et al. (2019) analyzed vHIT abnormality in terms of decreased vHIT gain and CSs occurrence in 3 patients with high-tone sensorineural hearing loss with vertigo and found that posterior SCC ipsilateral to side of hearing loss were severely impaired, while horizontal SCC and anterior SCC were preserved. Recently, several studies have investigated the association between vHIT results and hearing outcome in SSNHL patients with and without vertigo (Byun et al., 2020; Seo et al., 2022). Byun et al. reported that low VOR gain in posterior SCC had a specificity of 94.4% in predicting poor hearing outcome, suggesting that the decreased VOR gain of posterior SCC appears to be a specific prognostic factor for incomplete hearing recovery in SSNHL patients (Byun et al., 2020). Most recently, Seo et al. also demonstrated that decreased vHIT gain in posterior SCC was associated with the poor prognosis of hearing as well (Seo et al., 2022).

These previous findings and our results suggest that posterior SCC is preferentially vulnerable in patients with SSNHL, especially those with vertigo symptoms. The pathophysiological mechanism underlying this impairment pattern has not been fully elucidated. One possible explanation was disturbance of the vascular supply to the inner ear. Anatomically, the labyrinthine artery terminates in two major branches: the anterior vestibular artery (AVA) and the common cochlear artery (CCA), which are located at the bottom of the internal auditory canal. The AVA mainly supplies the ampulla of the anterior and horizontal SCC, the utricle macula, and the superior part of the saccular macula of the labyrinth, while the CCA supplies the cochlea, the posterior SCC, and the inferior part of the saccular macula (Kim et al., 1999). In patients with SHLV, cochlear and vestibular symptoms can manifest concurrently or successively. Considering that SHLV patients have the highest rate of pathological vHIT in posterior SCC, and that reduced vHIT gain of posterior SCC might be a specific prognostic factor for poor hearing recovery in SSNHL patients, it is reasonable to assume that vascular disturbance of the CCA, which co-supplies the posterior SCC and cochlea, might be involved in some SHLV patients. In a recent case with unilateral SHLV reported by Castellucci et al. (2020), a selective vHIT gain reduction of the ipsilateral posterior SCC has been documented, and filling defect in this SCC has been detected by inner ear MRI, which was thought to be fibrosis due to ischemia. These findings, from a radiological perspective, further suggested that the posterior SCC hypofunction caused by ischemia may be a characteristic pathological change in some SHLV patients. Nevertheless, isolated hypofunction of posterior SCC in AVS patients might suggest other underlying diagnoses. Castellucci et al. found that in 13 ongoing or previous AVS patients with selective posterior SCC loss, there were 3 cases with inferior vestibular neuritis and 10 cases with ISSNHL (Castellucci et al., 2021). Tarnutzer et al. found that the most frequent causes in 40 patients with unilateral isolated loss of posterior SCC were history of VN (13/40, 33%), Ménière’s disease (10/40, 25%) and vestibular schwannomas (7/40, 18%) (Tarnutzer et al., 2017). Therefore, the mechanisms of posterior SCC hypofunction in vestibular disease remain to be further explored.

### 4.2. The lesion patterns of vHIT in VN patients

The present study found that, among three SCCs in patients with VN, horizontal SCC had the highest rate of pathological vHIT results and the CSs occurrence, suggesting that horizontal SCC may be the most vulnerable SCC in VN patients. Magliulo et al. found that, in 40 patients with VN, 35 (87.5%) cases exhibited the decreased vHIT gain in horizontal SCC, 31 (77.5%) in anterior SCC, and 19 (47.5%) in posterior SCC (Magliulo et al., 2014). Furthermore, Taylor et al. showed that VOR gain reduction, measured by vHIT, were more prevalent in horizontal SCC (97.7%) and anterior SCC (90.7%) than in posterior SCC (39.5%). For otolithic function measured by VEMP, utricular dysfunction (72.1%) was more frequent than saccular dysfunction (39.0%) (Taylor et al., 2016). With respect to lesion pattern of SCCs measured by vHIT in patients with VN, our results were concordant with previous studies, showing that horizontal SCC is commonly affected, followed by anterior and posterior SCC.

It is generally believed that VN is associated with viral infection of vestibular nerve, which is subdivided into superior and inferior divisions. The superior division innervates horizontal SCC, anterior SCC and utricle, while the inferior division innervates posterior SCC and saccule. VN preferentially affects the superior division (Fetter and Dichgans, 1996). This may be attributed to the anatomical differences between these two nerves. The bony channel of superior vestibular nerve is seven times longer than that of the inferior vestibular nerve, and has a higher ratio of bony spicules occupying the canal in which the superior nerve and its arterioles course (Goebel et al., 2001; Gianoli et al., 2005), resulting in a higher incidence of mechanical or ischemic insults in the superior vestibular nerve. Thus, horizontal and anterior SCC, innervated by the superior vestibular nerve, are more likely to have pathological vHIT outcomes.

Our results also found that, in affected side of VN patients, the incidence of VOR gain reduction was highest in anterior SCC (90.32%), followed by horizontal SCC (80.65%) and posterior SCC (64.52%). No difference was found between anterior and horizontal SCC, and the difference was statistically significant between anterior and posterior SCC. Considering that the disease duration in our VN patients was relatively long (median = 20 days)，and most voluntary head movements in daily activities are in the horizontal plane，it is likely that vestibular compensation has already happened and the VOR gain has begun to recover. Previous longitudinal follow-up studies have shown that restoration of VOR gain in VN patients was faster than the saccades during vestibular compensation (Esteban-Sanchez and Martin-Sanz, 2022; Psillas et al., 2022), and saccades were most prevalent in horizontal SCC among the three SCCs during follow-up (Psillas et al., 2022).

### 4.3. Differences of lesion patterns between SHLV and VN patients in terms of vHIT findings

SHVL and VN, both manifested as AVS, could easily be differentiated based on cochlear symptoms. Our results showed that the incidences of pathological vHIT and CSs occurrence in anterior and horizontal SCCs in VN patients were significantly higher than those in SHLV patients. And the VOR gain in anterior and horizontal SCCs in VN patients was significantly lower than that in SHLV patients. Furthermore, no significant difference in vHIT results of posterior SCC was found between two groups. These findings suggested that, compared with SHLV patients, high frequency angular VOR in horizontal and anterior SCCs was compromised to a greater extent in VN patients, while posterior SCC function was comparable. Our results were in line with those reported by Yao et al., who found lower vHIT gain and higher asymmetry ratio of anterior and horizontal SCCs in VN patients, compared with SHLV patients, and no difference was observed in posterior SCCs between two groups (Yao et al., 2018). Furthermore, Jung et al. examined the extent of vestibular damage by using a comprehensive battery of vestibular function tests, and demonstrated a higher incidence of pathological cVEMP results in SHLV patients (76.9%) than in VN patients (42.3%) and comparable results with regard to horizontal SCC/superior vestibular nerve function. The authors suggested that SHLV patients are more prone to saccule/inferior vestibular nerve involvement (Jung et al., 2012). Preferential involvement of posterior SCC in conjunction with hearing loss in patients with SHLV could be better explained by vascular insult (Yao et al., 2018). On the other hand, isolated vestibular dysfunction without cochlear damage in the VN patients with functional loss in either the horizontal and anterior SCCs, or all three SCCs, is more likely to be caused by vestibular neuropathy. It is noteworthy that ischemic damage is also one of the possible etiologies for patients with isolated vestibular dysfunction without cochlear damage. The AVA is the smallest terminal branch of the internal auditory artery and lack collateralizations (Grad and Baloh, 1989; Kim et al., 1999). The territory it supplies is mostly in line with which the superior vestibular nerve innervated (Mazzoni, 1990; Kim and Lee, 2009). Therefore, ischemic damage in this branch could result in hypofunction of anterior and horizontal SCCs, which is consistent with the superior VN (Comacchio et al., 2021). Similarly, the posterior vestibular artery supplies the posterior SCC and saccule (Kimura and Perlman, 1958a,b; Mazzoni, 1972). Therefore, sometimes it is difficult to distinguish the ischemia in this branch from the inferior VN. Comacchio and Castellucci recently reported a case with AVS mimicking inferior VN developed posterior SCC ossification on follow-up, which might be attributed to the ischemia of posterior vestibular artery (Comacchio and Castellucci, 2022). It indicates that the etiology of VN is still in dispute and the pathophysiological differences between SHLV and VN patients remain to be further elucidated.

We also found a significantly higher CP value in VN group than in SHLV group, suggesting that low-frequency angular VOR function in horizontal SCC represented by caloric response was more severely damaged in VN, which coincided with the vHIT findings in horizontal SCC in our series.

### 4.4. Strength and limitation

The main strength of our study is that CSs and VOR gain in vHIT was jointly used in assessing the VOR function in SCCs. vHIT results can be affected by a variety of confounding factors (Matino-Soler et al., 2015; Patterson et al., 2015; Kim et al., 2018; van Dooren et al., 2020). It has been reported that interpreting vHIT using gain and the presence of a CS could improve the diagnostic accuracy of vHIT for identifying vestibular loss (Janky et al., 2018; van Dooren et al., 2020). Recently, Yang et al. found that the abnormal rates based on vHIT gains alone or on both vHIT gains and CS measurements were similar at the acute stage but were significantly higher when considering both vHIT gains and CS compared with vHIT gains alone at the follow-up stage (Yang et al., 2018). As the course duration in our VN group was significantly longer than that in SHLV group, vHIT analysis based on both gain and CS could reflect the severity of vestibular impairment more accurately compared with using the vHIT gain alone.

Our study has several limitations. First, the duration of the disease is significantly longer in patients with VN than in patients with SHLV. Patients with VN were more likely to present first for care to the neurology or emergency department due to absence of auditory symptoms, thus longer time had elapsed before they were referred to our clinic. Future studies should be prospectively conducted to include patients with VN and SHLV with comparable course duration. Secondly, no follow-up data was collected due to the cross-sectional design of this study. Several studies have demonstrated that, in the chronic stage, the residual symptoms of VN were associated with the outcomes of vHIT. Lee and Kim found that the symptoms-free VN patients had higher vHIT gain than those with residual symptoms (Lee and Kim, 2020). During follow-up, Fu et al. showed that although the incidence of overt saccades was significantly reduced (100% in the acute stage, 59.58% in the follow-up), covert saccades could still be detected in a large proportion of VN patients (100% in the acute stage, 87.23% in the follow-up) (Fu et al., 2019). Thirdly, we lacked results in VEMPs herein. A combination of vHIT and otolith test (cervical and ocular VEMP) allows a comprehensive assessment of all labyrinthine end-organs and afferents, enabling accurate mapping of peripheral vestibular dysfunction and the identification of specific lesion patterns (Curthoys, 2012). Therefore, prospective studies with long-term follow-up and thorough neurotological evaluations including vHIT and VEMPs are needed in future to further explore the mechanisms of vestibular lesion pattern in these two vestibular disorders.

## 5. Conclusion

In this study, vHIT analysis was based on both gain value and CSs. SHLV patients were more likely to have posterior SCC impairments, while VN patients were more prone to horizontal and anterior SCC injury, which implied that these two diseases presenting as AVS may have different pathophysiological mechanisms.

## Data availability statement

The original contributions presented in the study are included in the article/supplementary material, further inquiries can be directed to the corresponding authors.

## Ethics statement

The studies involving human participants were reviewed and approved by Ethical Committee of Union Hospital, Tongji Medical College, Huazhong University of Science and Technology, Wuhan, China. The patients/participants provided their written informed consent to participate in this study.

## Author contributions

YZL: interpretation of data, statistical analysis, and drafting of the manuscript. YML: patient consultation, interpretation of data, and critical revision of the manuscript. RZ, JL, and HW: patient recruitment and data collection. KX: patient recruitment, data collection, and statistical analysis. HX: study conception and design, interpretation of data, and critical revision of the manuscript. BL: study conception and design, patient consultation, interpretation of data, and critical revision of the manuscript. All authors contributed to the article and approved the submitted version.

## Funding

This work was supported by grants from the National Natural Science Foundation of China (No. 81670930), the Natural Science Foundation of Hubei Province, China (No. 2021CFB547).

## Conflict of interest

The authors declare that the research was conducted in the absence of any commercial or financial relationships that could be construed as a potential conflict of interest.

## Publisher’s note

All claims expressed in this article are solely those of the authors and do not necessarily represent those of their affiliated organizations, or those of the publisher, the editors and the reviewers. Any product that may be evaluated in this article, or claim that may be made by its manufacturer, is not guaranteed or endorsed by the publisher.
